# A new instrument for the assessment of laterality: evidence from confirmatory analysis

**DOI:** 10.1007/s10072-025-08164-0

**Published:** 2025-04-17

**Authors:** Mirko Duradoni, Andrea Frosini, Giorgio Gronchi, Andrea Peru

**Affiliations:** 1 Department of Education, Language, Literature and Psychology, Università degli Studi di Firenze, Via di San Salvi, 12 Pad. 26-50135, Firenze, Italy; 2 Department of Mathematics and Computer Science “Ulisse Dini”, Università degli Studi di Firenze, Viale Morgagni, 67/a - 50134, Firenze, Firenze, Italy; 3 Department of Neurosciences, Psychology, Drug Research and Child Health, Università degli Studi di Firenze, Via di San Salvi, 12 Pad. 26-50135, Firenze, Italy

**Keywords:** Laterality, Questionnaire, Hand, Foot, Eye, Ear preference, Cognitive organization, Cognitive assessment

## Abstract

Despite its importance, the assessment of laterality in scientific and clinical contexts remains inconsistent. Many studies rely on self-reports or outdated questionnaires (in terms of daily-related actions), often neglecting lateralization of the lower limbs, eyes, and ears without considering the four effectors at the same time. To address these limitations, we developed the Florence Laterality Inventory (FLI), a 16-item scale designed to provide a comprehensive assessment of hand, foot/leg, eye, and ear preference through more contemporary and relevant questions. Our study, conducted with 225 participants, confirmed a four-factor structure through confirmatory factor analysis (CFA), demonstrating good reliability for the hand, eye, and ear subscales and, to a lesser extent, also for foot/leg. Correlations with established measures of laterality and handedness supported the concurrent validity of the scale. The FLI scale offers an updated multidimensional instrument to measure lateralization, addressing the shortcomings of previous questionnaires and adapting to modern contexts.

## Introduction

It is a generally accepted scientific principle that the individual’s behavioural and cognitive performance is influenced by demographic factors as age, gender, and education. Accordingly, information about these factors is usually provided in detail when research involves human subjects. Very often, however, information about subjects’ handedness– that is, the peculiar human tendency to prefer the right or left hand for fine manual tasks, such as writing, drawing and using tools - is also provided.

While the mechanisms by which age, gender, and education can modulate the individual’s cognitive functioning are obvious, the relationship between handedness and cognitive performance is more complex and, in some ways, more intriguing. It has mainly to do with the localization of language, and other cognitive functions, in the brain; namely, laterality. In the following, after a summary of the main debates related to handedness and laterality, we will present a preliminary study aimed at creating a tool to assess the laterality of upper limb, lower limb, eye, and ear.

## Handedness and laterality

Notwithstanding that the classic hemispheric dominance theory claiming that the localization of language (and other cognitive functions) in the left or right hemisphere correlates to handedness according to the principle of “cross-lateralization”, proved to be fallacious, consistent evidence suggests that some relationship between handedness and language laterality does exist (but see below for a different view).

The traditional doctrine, based on evidence from acquired language deficits [1, 2] and the selective suppression of activity in one cerebral hemisphere using sodium amytal [3], has claimed that in almost all right-handed individuals, language is localized in the left hemisphere, as it is in most individuals who are not right-handed. These findings have been substantially confirmed by more recent studies using functional magnetic resonance imaging [4, 5] or functional transcranial doppler [6].

However, while right-handed people (nearly 90% of the world’s population) represent a quite homogeneous group, the same cannot be said for those who are not right-handed. These people are collectively termed as adextrals and include individuals who show no preference (i.e., ambidextrous) and individuals who show a moderate to strong preference for the left hand (i.e., left-handed).

Such a heterogeneity reflects on the relationship between hand preference and language laterality, thus making the location of language in adextrals largely unpredictable. More generally, it is commonly thought that the cognitive organization and the localization of certain cognitive processes are more variable amongst adextrals.

Therefore, the presence of adextrals in current neuroimaging research is a rather rare occurrence: only around 3-4%; i.e., noticeably less than the 10-13% one would expect if neuroimaging samples were truly representative of the whole human population [7]. Roughly speaking, according to the anecdote reported by the authors themselves, “…if you weren’t right-handed, you weren’t going anywhere near a scanner” [7].

Maybe things would have been different, if you had accepted the idea that concordance of hemispheric dominance for hand and for language production occurs by chance with the sole exception of a very small group of individuals who show strong right hemisphere dominance for both language and hand control; namely, they are left-handed with language areas in the right hemisphere [8].

## Measuring handedness and laterality

Whatever it may be the link between handedness and cognitive organization, there is no doubt that, especially in the field of neuropsychology, the knowledge of the preferred hand of subjects involved in a study is often required. Not surprisingly, a survey on one randomly chosen annual volume of the two historical journals in the field (i.e., Neuropsychologia– founded in 1963– and Cortex– founded the year after) demonstrated that the subjects’ handedness is reported in about half of the published papers.

However, quite different is the criterion according to which the subjects’ handedness is determined in different studies. A direct assessment of hand strength and/or ability is found only in papers that specifically address that issue. Actually, in the majority of the other papers, participants are simply reported as right- or left-handed, based on their self-report. Finally, in a minority of cases, handedness is assessed by means of a properly devised questionnaire [9].

From a historical point of view, the interest for dextrality and sinistrality of hand (and eye) dates back to Galton’s studies [10]. However, the use of questionnaires to define manual preference became systematic in the years around World War II [11]. In the following decades, several instruments have been devised, starting with Annett’s [12] which then, in turn, inspired the development of further instruments. Among the most appreciated and used questionnaires, the following deserve to be mentioned: the Provins and Cunliffe’s questionnaire [13]; the Briggs and Nebes’s questionnaire [14]; the Handedness Questionnaire Cambridge [9]; the Stanley Coren’s Lateral Preference Inventory [15]; the Waterloo Handedness and Footedness Questionnaire-revised [16, 17], etc. Some of these questionnaires contain many items (e.g., Provins and Cunliffe’s questionnaire: 31 items; Waterloo Handedness and Footedness Questionnaire - r: 36(39) + 10(13) items), some of which concern activities that can be considered anachronistic. For this reason, some of these questionnaires have been recently revised. This is the case, for example, of the Provins and Cunliffe’s questionnaire revised by Nicholls and co-workers and now named FLANDERS [18]. Furthermore, the different questionnaires vary in their range of response formats: some require participants to respond along a three-point scale (left-either-right), while others use a five-point scale (always left, usually left, no preference, usually right and always right).

Despite such a wide range of choice, in the vast majority of papers, the questionnaire used is the Edinburgh Handedness Inventory [19] where participants have to indicate their preferred hand in a series of 10 daily live activities.

Although the worldwide popularity of this instrument (almost 11000 citations in Scopus) should guarantee an extreme reliability of data collected, also in this case, the activities (e.g., striking a match) and the items (e.g., brooms) involved are somewhat dated and young people do not have enough familiarity with them and, inevitably, things will get worse from here on out. For instance, in their research addressing the relationship between hemispheric lateralisation for language and handedness, Mazoyer and co-workers [8] presented participants with the Edinburgh Handedness Inventory, but dropped the “broom” item since young people had never used that tool. Moreover, for each item, the original questionnaire contemplates only two response options (i.e., left vs. right hand), although it is possible for the participants to emphasize those activities that they “…would never try to use the other hand unless absolutely forced to” by assigning two “++” marks to the preferred side as well as those for which they are “… really indifferent” by marking both sides with a “+”. Lastly, the instructions for the original Edinburgh Handedness Inventory were far from clear, so that they are usually misunderstood by most respondents [20].

To try and overcome such limits, a 7-item revised version of the original inventory has been proposed [21]. The new instrument was administered using the same “somewhat lengthy and confusing” instructions of the original version, thus making the findings questionable [20]. Approximately in the same period, Veale [22] administered the Edinburgh Handedness Inventory using simplified instructions and response options. A confirmatory factor analysis allowed the author to remove some items to obtain a 4-items revised version of the questionnaire that demonstrated to have adequate fit to measure a single handedness factor.

However, the research was part of a larger study designed to examine the development of gender identity and sexuality, and the analyses were run on 160 biological males and 266 biological females the vast majority of which were white Caucasian living in only one geographic region (i.e., USA). Even more importantly, the questionnaire did not assess the preference for lower limb, nor for the eye or the ear.

It is well established that handedness and footedness do not measure the same construct, thus indicating substantial independence of hand preference and foot preference [23], as well as of eye and ear preference. Even more importantly, when each lateral preference is considered by itself, handedness and, in a lesser extent, footedness but not eyedness and earedness are somewhat predictive of the hemispheric specialization for language. However, such a predictive value in assessing cerebral language dominance, increases when the degree of congruency across indexes (all right, all left, mixed) is considered with a left-sided bias on all four indices (hand, foot, eye, ear) that was likely to occur with right, but not left, hemisphere language representation whereas right-sided congruency was typical of left, but not right, hemisphere speech dominance [24].

Hereafter, we report the findings of a study properly devised to develop a new short questionnaire aimed to measure the lateralization of the four effectors with items that describe more common actions today compared to previously published scales.

## Methods and procedure

### Instrument

The initial version of the questionnaire (called FLI, Florence Laterality Inventory) consisted of 17 items: 8 for the hand and 3 for foot, eye, and ear, respectively. All the items except one were a new formulation of items originally proposed in various questionnaires. Table 1 shows, for each item, the item from already existing questionnaires that inspired it. Response format was a 5-point Likert scale from “Absolutely Left” to “Absolutely Right”). Participants were asked to complete the questionnaire without any time constraints. The procedure usually took no more than ten to fifteen minutes.

**Table 1:** City laterality inventory– list of the items

Item	Taken from / Inspired by
Hand Items: Which hand do you use when employing it…	
1. To throw a ball to hit a target	Waterloo Handedness Questionnaire rev: Item 5
2. To insert coin into the appropriate slot	Waterloo Handedness Questionnaire rev: Item 22
3. At a party to hold the dish while picking up a glass of beverage	Original
4. To rub writing off a blackboard (with an eraser or a selvedge)	Waterloo Handedness Questionnaire rev: Item 16
5. To hold a fruit (potato) while peeling it	Brief Handedness Questionnaire: Item 9
6. To stir a liquid with a spoon	Waterloo Handedness Questionnaire rev: Item 30
7. To put lip balm on your lips	Waterloo Handedness Questionnaire rev: Item 24
8. To hold a pocket-knife while opening the blade	Handedness Questionnaire Cambridge 1977: Item 22
Leg/Foot Items: Which leg/foot do you use when employing it…	
1. To stand on one foot	Waterloo Footedness Questionnaire rev: Item 2
2. To take a leap forward/high	Waterloo Footedness Questionnaire rev: Item 8
3. To climb the first step of a ladder	Waterloo Footedness Questionnaire rev: Item 4
Eye Items: Which eye do you use when employing it…	
1. To look down a microscope/magnifying glass	Handedness Questionnaire Cambridge 1977: Item 1
2. To look through a keyhole	C Handedness Questionnaire Cambridge 1977: Item 3 C
3. To take aim at a target with a rifle/bow (eye open)	Stanley Coren Questionnaire: Item 12
Ear Items: Which ear do you use when employing it…	
1. To find out if a clock is ticking	Stanley Coren Questionnaire: Item 16
2. To hear someone’s heartbeat	Stanley Coren Questionnaire: Item 15
3. To listen in on a conversation going on behind a closed door	Stanley Coren Questionnaire: Item 13

### Sample and sampling

For the scope of our study, it is pertinent to note that a minimum recommended ratio of 10 participants per item was adhered to, as suggested by previous research [25]. Moreover, a sample size equal to or greater than 200 individuals was considered a *fair* prerequisite for conducting confirmatory factor analysis, as per established guidelines [26, 27]. Given that our dataset comprised responses from 225 participants, we confidently assert that the sample size for our study was unequivocally sufficient. Of the 225 participants 64.9% were female, and the average age of the sample was 34.90 (s.d. = 13.45; age range = 14–74).

### Data analysis

We conducted a confirmatory factor analysis using the AMOS software to assess the dimensionality of the scale. For parameter estimation within the model, we employed maximum likelihood estimation (MLE). The evaluation of model fit relied on a comprehensive set of goodness-of-fit indices, including the Chi-square to degree of freedom ratio (χ2/df) [28], the Tucker–Lewis index (TLI) [29], the standardized root mean square residual (SRMR) [30], the root mean square error of approximation (RMSEA) [31], and the comparative fit index (CFI) [32]. To gauge model adequacy, we considered specific thresholds: a TLI value exceeding 0.95, a CFI value approximating 0.95 (with a range of 0.90 to 0.95 indicating a good fit), an SRMR value below 0.08, and an RMSEA value less than 0.06 (with 0.06 to 0.08 signifying a favourable fit) [33].

We assessed the reliability of the test using McDonald’s omega, a preferred choice in psychometric literature due to the limitations of Cronbach’s alpha [34–36]. Nevertheless, as Cronbach’s alpha can be considered a special case of omega under specific conditions [37], we applied the interpretation guidelines for alpha to discuss the reliability of our scale. To clarify, we categorized Cronbach’s alpha values as minimally acceptable (α = 0.65), acceptable (α = 0.70), and optimal (α ≥ 0.80) [38]. Finally, to assess the consistency and reproducibility of results obtained (test-retest reliability), we re-administered the FLI to a small subgroup of participants (i.e., 40 young adults) over six months from the first administration. The scores from the second administration were absolutely consistent with those from the previous one (0.95, 0.84, 0.89 and 0.86 for hand, foot/leg, eye, and ear preference, respectively), thus confirming the good reliability of our instrument.

## Results

### Confirmatory factor analysis and reliability

**Fig. 1:**
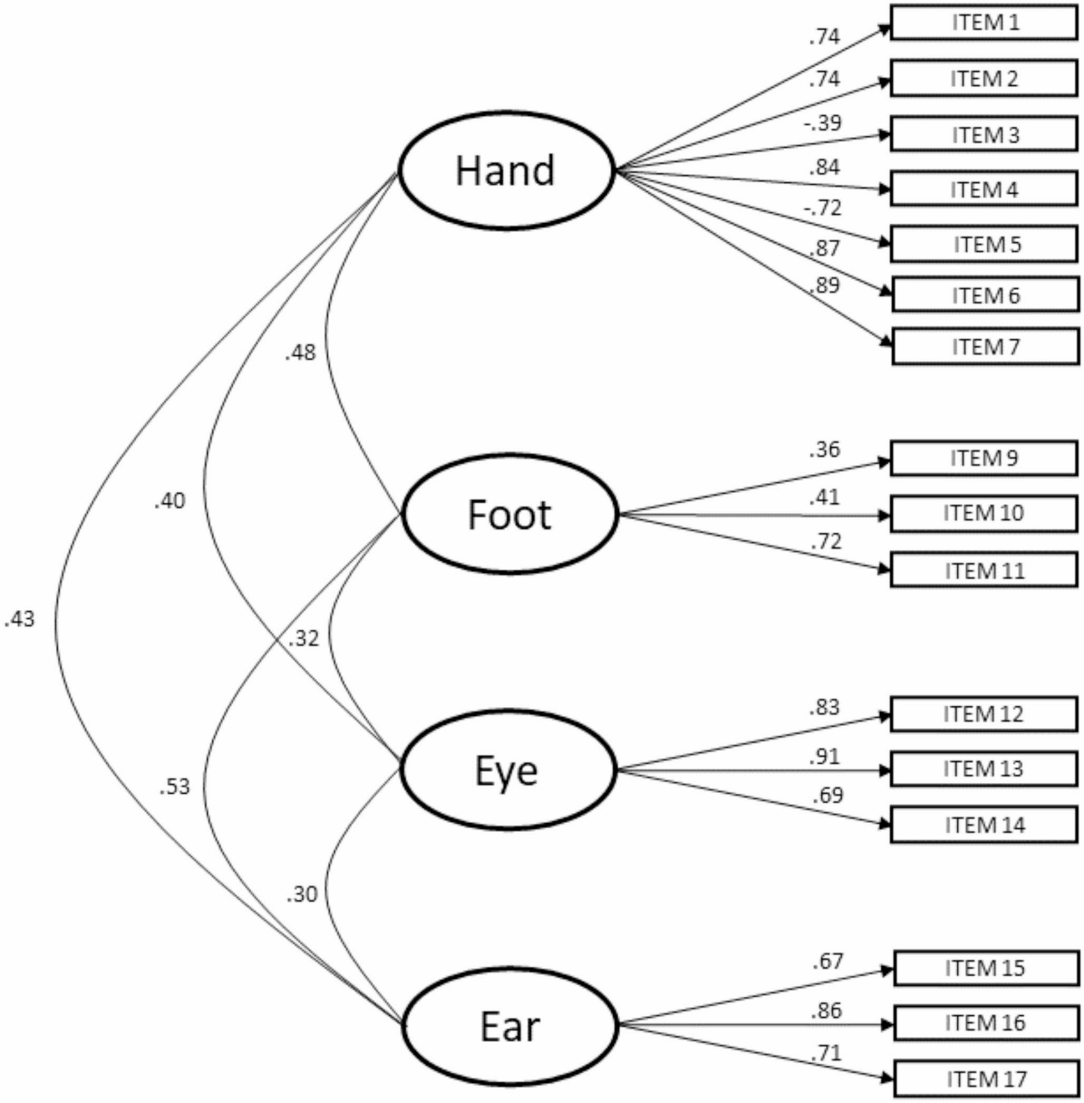
Results of Confirmatory Factor Analysis

CFA was performed to test the hypothesized 4-factor structure on the 17 items. Despite quite remarkably fit indices (χ2/df = 1.63; p.<0.001; TLI = 0.95; CFI = 96; RMSEA = 0.05; SRMR = 0.06), one of the items, namely item 8, displayed a non-significant factor loading and was thus removed from the analysis. The removal of item 8, increased measurement model fit to the data (χ2/df = 1.54; p.<0.001; TLI = 0.96; CFI = 97; RMSEA = 0.05; SRMR = 0.05). Overall, CFA appeared to support the supposed 4-factor structure for the scale as shown in Fig. 1.

As for reliability, these are the McDonald’s omega values for upper limbs, lower limbs, eye, and ear respectively: ω = 0.90; 0.50; 0.85; 0.80.

### Concurrent validity

As a first step, we computed the total score for each dimension of our scale based on the CFA identified solution. Items 3 and 5 were reverse scored before summing them with the others pertaining to the upper limb dimension.

Before proceeding with the inferential analyses to test validity, we produced descriptive statistics assessed variables normality’s (asymmetry and kurtosis values). As we can gather from Tables 2, 4 variables did not appear to be normally distributed. For this reason, we opted to analyse data through non-parametric statistics, namely Spearman’ Rho and Kruskal-Wallis ANOVA.

**Table 2:** Descriptive statistics of the City laterality inventory scale and concurrent validity measures

Variables	Min	Max	Mean	s.d.	Skewness	Kurtosis
Hand	8	35	28.68	6.29	-1.81	2.94
Leg/Foot	3	15	10.37	2.32	-0.63	0.75
Eye	3	15	10.51	3.38	-0.54	-0.53
Ear	3	15	10.05	2.51	-0.59	0.91
Look with one eye	1	5	3.26	1.32	-0.35	-0.95
Listen with one ear	1	5	3.37	1.00	-0.22	0.10
Laterality quotient	-100	100	62.44	54.24	-1.92	2.63
Waterloo Handedness	-52	72	36.05	26.52	-1.69	2.72
Waterloo Footedness	-16	20	7.53	6.86	-0.82	1.33

Correlation analysis (Table 3) supported the concurrent validity of our scale. The FLI was correlated with a 10-item version of Edinburgh Handedness Inventory (Laterality Quotient in the tables), the Waterloo Handedness Questionnaire, and the Waterloo Footedness Questionnaire. Also, two ad hoc items (“Look with one eye”; “Listen with one ear”) were included for eye and ear preference. Each variable dedicated to measuring the laterality of a specific body part correlated substantially with the respective dimension of the FLI scale. The dimension related to hand correlates more strongly, as expected, with the Laterality Quotient and Waterloo Handedness. The dimension pertaining to leg/foot positively correlated with Waterloo Footedness, while the dimensions related to eye and ear laterality positively correlated with their respective ad-hoc items.

**Table 3:** Correlation analysis between the City laterality inventory scale dimensions and concurrent validity measures. Note: ^***^= P.<0.001; ^**^= P.<0.01; ^*^= P.<0.05

Variables	Laterality Quotient	Look with one eye	Listen with one ear	Waterloo Handedness	Waterloo Footedness
Hand	0.54^***^	0.15^*^	0.21^***^	0.67^***^	0.41^***^
Leg/Foot	0.25^***^	0.01	0.29^***^	0.26^***^	0.60^***^
Eye	0.16^*^	0.68^***^	0.34^***^	0.20^***^	0.19^**^
Ear	0.34^***^	0.27^***^	0.67^***^	0.44^***^	0.44^***^

Since Laterality Quotient provides the opportunity to cluster participants into “Left-handedness”, “Ambidexterity”, and “Right-handedness” based on score cut-offs, we compared these categories based on our 4 dimensions. For all of them, we observed and increase in mean rank passing from Left-handedness to Right-handedness (Hand: χ2 = 59.29; p.<0.001; Leg/foot: χ2 = 13.04; p.<0.001; Eye: χ2 = 29.14; p.<0.001; Ear: χ2 = 18.88; p.<0.001).

## Discussion

Although not yet fully understood, there is no doubt that there is some relationship between handedness and cortical organization, with a particular emphasis for the localization of language centres in the brain. While the picture is highly consistent among right-handed individuals with language localized to the left hemisphere in virtually all of them, the issue becomes much more complicated when considering individuals who are not right-handed.

It is certainly true that– consistently with the relationship found in right-handed people– also in most of the individuals who are not right-handed, collectively defined as adextrals, language is localized in the left hemisphere so that some authors argue that these groups are effectively homogeneous with respect to cerebral asymmetry and the effects of handedness on hemispheric specialization for language are scarcely relevant [39, 40]. However, a recent, exhaustive, meta-analysis demonstrated a consistent, albeit subtle, left hemispheric dominance in right-handed individuals compared to adexrals [41].

Another aspect complicates the issue. While right-handed individuals represent a fairly consistent group where almost everybody shows a strong preference for the right hand for fine manual tasks, adextrals represent an extremely heterogeneous group that includes ambidextrous, and from moderate to strong left-handed. This is particularly relevant considering that, regarding cognitive performance, the degree of manual dexterity appears to be a better predictor than direction [42].

For the above reasons, it is clear the need for a fast and reliable procedure for the assessment of manual preference for both clinical and scientific purposes. To this aim, we have developed the FLI. The CFA results verified the 4-factor structure with optimal reliability of each subscale except for leg/foot. In line with expectations, items corresponding to the four modalities (hand, leg/foot, eye, ear) have been grouped into four different factors.

In proposing this scale, we aimed to update classic items from old tools by referring to more commonly performed actions that involve all four effectors. As evidenced by the results, this approach has yielded satisfactory outcomes. The only item that was eliminated was “To hold a pocketknife while opening the blade”. Its removal led to a significant increase in the model’s fit. We can hypothesize that this is because it is a less common action compared to those described by the other items.

Regarding the reduced reliability of the subscale of the leg/foot, it seems likely that it may depend on the fact that the activities of the lower limb are much more gross than those of the hand, eye, and - to some extent - even the ear. In this way, the fine motor skills required to perform all the activities tested in the hand questions as well as the fine sensory discriminations at work in the eye and ear tasks, lead to a strong and consistent preference for a well-defined effector. Otherwise, individuals perceive their lower limbs more interchangeable and tend to choose responses like “preferably with” rather than “always with”, this resulting in a less pronounced preference for lower limb effectors.

As for the external validity of the scale, each variable aimed at assessing the laterality of a specific body part displayed a significant correlation with the relevant dimension of the FLI scale. Predictably, the dimension linked to hand showed a stronger association with both the Laterality Quotient and the Waterloo Handedness Questionnaire. Likewise, the dimension associated with lower limbs exhibited a positive correlation with the Waterloo Footedness Questionnaire. Additionally, the dimensions for eye and ear laterality were positively correlated with their respective ad-hoc items. These findings suggest that the laterality measures for each body effector are reliable and align well with established laterality assessments.

The necessity of a new questionnaire relies on the usefulness of such kind of measure (i.e., self-report is poorly reliable whereas the direct assessment of hand/leg strength and/or ability is unpracticable for reasons of time and costs) and the limitations of the scales currently present in literature. Indeed, the Edinburg Inventory, considered the golden standard in the field, has been criticized for obsolete item [8], and it turned out that its instructions are likely to be misunderstood by respondents with fewer years of formal education [20]. The shortened version of the questionnaire recently developed [21] has been criticized because of unclear instructions. Veale and co-workers [22] further revised and shortened the scale which now consists of only 4 items. Although validated by a confirmatory factor analysis, findings from this questionnaire only refer to the preferred hand, leaving the issue of foot eye and ear preference completely unaddressed. There is no doubt that any discussion on laterality must also include the preference for the eye as well as for the lower limb and ear. Few questionnaires addressed these issues. The Stanley Coren’s Lateral Preference Inventory [15] consists of 12 items: 4 items each for handedness, footedness, eyedness and earedness. Some items refer to activities now viewed as anachronistic; furthermore, many of them are formulated in a too convoluted way (e.g., “*Imagine a small box resting on a table. This box contains a small clock. Which ear would you press against the box to find out if the clock was ticking?*”; “*If you wanted to pick up a pebble with your toes*,* which foot would you use?*”). The same criticisms can be made to the footedness section of the Waterloo questionnaire (10 + 3 items); actually, it includes the 4 items of the Coren inventory, formulated, if possible, in an even more convoluted form and another 6 items concerning activities that are now little practiced (e.g. “*Which foot would you use to help push a shovel into the ground*?”).

It is important to highlight the limitations of this work, which represents only a first step in the development of a comprehensive tool. First, it is necessary to replicate the stability of the factorial structure in a future study, considering a sample stratified by age and gender to highlight potential differences. Even more importantly, it is necessary to verify the external validity of each subscale with a behavioural criterion test (e.g., a foot tapping test). Finally, it would be beneficial to explore the neural correlates of the FLI to determine whether the laterality of different effectors aligns with left-hemisphere specialization for language.

In sum, it seemed to us that the time was ripe for a new, carefully validated instrument that could provide reliable insight on individual preference for each of the 4 systems: hand, foot, eye and ear with more simple, easy to understand and imagine actions. We hope that the data collected in this paper could represent a first step in the development of a new laterality scale.
